# Fractal Analysis in Pore Size Distributions of Different Bituminous Coals

**DOI:** 10.1038/s41598-019-54749-z

**Published:** 2019-12-03

**Authors:** Jie Zhu, Fa He, Yang Zhang, Rui Zhang, Bo Zhang

**Affiliations:** School of Mechanics & Civil Engineering, China University of Mining & Technology (Beijing), Beijing, 100083 China

**Keywords:** Geodynamics, Geodynamics, Geodynamics, Mineralogy, Mineralogy

## Abstract

Coal bumps, coal and gas bursts are currently the main threats to coal mine safety in China. The physical properties of coal are important determining factors for the occurrence of coal bumps or coal and gas bursts. A series of experiments using mercury intrusion porosimetry (MIP), nitrogen adsorption (NA) and carbon dioxide adsorption (CA) were employed to investigate the pore size distributions (PSDs) of bump-prone coal and gas-outburst coal. Considering the influence of coal matrix compressibility on the MIP experimental data, the MIP data should be considered in combination with NA and CA testing data. The dominant pores of gas-outburst coal are different from those of bump-prone coal. The PSDs of coal samples have multifractal characteristics. However, the multifractal characteristics of two types of coal are different. (Answer to question 1, reviewer 2). A comparison of the multifractal parameters indicated that Xin Zhou Yao (XZY) coal samples have a higher spatial heterogeneity and complexity of their pore size distribution, while Zhao Ge Zhuang (ZGZ) coal samples have a lower heterogeneity and pore connectivity, which may hinder smooth gas flow and lead to a localized collection of gas in coal seams.

## Introduction

Coal is the main energy source in China. With increases in the exploiting depth and intensity of coal mining in China, the occurrence of dynamic disasters, such as coal bumps or coal and gas outbursts, is increasingly frequent^1^. Underground mines have long experienced sudden, usually unexpected, expulsions of coal and gas away from freshly exposed working faces during underground mining. These are commonly known as coal and gas outbursts^2^. In general, a coal bump refers to a sudden and violent failure of a coal seam that releases the contained elastic energy and expels a large amount of coal and rock into the roadway or working face, while the participation of gas is ignored^3^. The dynamic disasters are a nonlinear dynamic process in which the steady state system energy accumulates and the unsteady state system energy is released in coal mines with specific geological characteristics. Further, such disasters are a result of coupled multiphysical fields including the external load environment, internal structure, textural structure and physical and mechanical properties.

Coal has a multi-pore structure that includes different parameters such as specific surface areas (SSAs) and pore size distributions (PSDs), which characterize the pore network inside a coalbed and is an important daily resource providing energy industrially and commercially^4^. However, the complex pore structure of coal, which is a multi-porous material, is sensitive to storage gas, and it has been found to be necessary to investigate the pore structures inside coal. The pore structure not only influences the gas reserving ability but also affects gas transporting^5^. Many researchers have paid attention to the mechanism of dynamic coal mine disasters as it relates to the macroscopic mechanical characteristics of coal rock, while only a few researchers have focused on the influence characteristics of coal microstructures on dynamic disasters. The pores in the material have a great influence on the properties of that material. Asit Kumar Gain^6^ found that the pore morphology, compressive strength and elastic modulus could be changed dramatically by increasing the pore forming agent. Combining the microscale seepage of the circular tube model and the seepage theory of the pore size effect, Jian Chen^7^ demonstrated that the seepage behavior of low-permeability clays is influenced by the pore size distribution effect. For rock materials, A. Fakhimi’s^8^ research results show that in addition to porosity, the pore size can have drastic effects on the elastic modulus, crack initiation stress and rock strength. (Answer to question 2, reviewer 2). Asit^9^ elucidated the microstructural changes of Sn-3.5 wt% Ag, an electronic interconnected material, after exposure to high temperatures and relative humidity (85 °C/85%). Such microstructural changes negatively impact material properties such as the electrical resistivity, elastic and shear moduli, hardness and creep performance. Based on the results of Dong Gyu Lee’s study^10^, it is possible to more easily predict the material properties of ceramic composite materials by the porosity and pore dispersity or adjacency.

To investigate the pore structure of coals, a variety of effective methods such as mercury intrusion porosimetry (MIP), small angle X-ray scattering (SAXS), gas adsorption analysis (GAA), scanning electron microscope (SEM) and computed tomography (CT)^11–16^ have been successfully applied. Although these methods allow the pore size distribution to be obtained quantitatively, there is hardly an effective approach to calculate and conclude the data from the above methods for coal, which is a multi-porous material. Nevertheless, the complexity of the pore structure and the impact of the pore structure on dynamic disasters cannot be easily described. The fractal method was initially proposed by Mandelbort in 1967, and the fractal dimension obtained from the fractal method has been widely used in qualitatively characterizing the physical properties of heterogeneous materials^17^. The two most frequently used methods are the singular fractal method and the multiple fractal method^18,19^. The fractal method was first applied to describe the pore structure of coals by Gauden^20^. By combining the fractal methods with the pore structure data from those above methods, it is comparatively easy to quantitatively characterize the pore structure. As time goes by, different fractal dimensions have been proposed and developed as better descriptions to produce more harmonic results and figures. The fractal dimensions are also divided into the surface dimension (*D*s) together with the volume fractal dimension according to different research purposes, mathematical equations and algorithms for which they are used during transforms. Generally, the body fractal dimension is usually used to describe the pore structure, and the surface dimension is better for calculating the specific surface area and characterizing the pore surface. Sun *et al*. described two surface dimensions, *D*_FHH_^21^ and *D*_SN_^22^, which were used to analyze the adsorption capability between shallow and deep deposited coals^23^. Li *et al*. used mercury injection porosimetry to analyze the compressibility and the pore structure^24^. In combination with MIP data and N_2_ and CO_2_ adsorption data, Li analyzed the pore porosimetry based on different PSDs of three kinds of coals, the multifractal method was used to characterize the pore categories and the generalized spectra showed how to describe the differences among the different PSDs of coal^25^. Caniego *et al*. used the singular multifractal method to analyze the pore size distribution in soil samples^26^. The coal pore characteristics in different dynamic coal mine disasters have been preliminary analyzed^27^.

To better understand the pore structure of coal samples from the 9# coal seam (coal and gas outburst seam) in the Zhao Ge Zhuang mine and the 11# coal seam (bump-prone coal seam) in the Xin Zhou Yao mine, the MIP method and N_2_ adsorption experiments were performed. By analyzing the data and noting the differences of the coal samples from different coal seams, this study calculates the multifractal singularity spectra and the generalized dimension spectra, which are significant parameters in characterizing the pore structure and important links between PSDs and dynamic disasters.

## Coal Sample Background

Six of the blocks were from the 11# coal seam of the Xin Zhou Yao mine in China, and 15 coal-rock burst accidents have occurred at working face 8937 in this coal seam. Two coal bumps in the 11# coal seam happened in October 2013 and March 2014. According to the method for the determination of coal seam impact tendency classification and index (China), the 11# coal seam of the Xin Zhou Yao mine is a bump-prone mine. The other 6 cubic coal blocks were drilled from working face 3197, 9# coal seam in the Zhao Ge Zhuang mine in China. The maximum gas pressure of the 9# coal seam is 2.71 MPa, and the gas content in the 9# coal seam is 6.6–8.7 m^3^/t. A total of 17 gas outburst accidents have happened at the 9# coal seam in the Zhao Ge Zhuang mine. According to the Provisions on Prevention and Control of Coal and Gas Outbursts (China), this seam is gas-outburst prone. And the coal is bituminous coal. Twelve coal blocks were collected during the mining process at working face 3197, 9# coal seam in Zhao Ge Zhuang mine and working face 8937, 11# coal seam in Xin Zhou Yao mine. We drilled cylinder coal samples (50 ∗ 100 mm) along bedding plane direction from each block for uniaxial compression experiment. Fragments with size less than 1 cm ∗ 1 cm ∗ 1 cm close to strain sensors were collected for MIP, NA and CA experiments. Therefore these coal samples used for MIP, NA and CA testing are thought to be representative. (Answer to question 2 reviewer 1 and editor).

During coal block drilling process, we collected coal fines and sieved the fines through a screen with a diameter of less than 1 mm. The coal fines were sealed in a plastic bag before proximate petrographic composition analysis. The proximate analysis and maceral of different samples are shown in Table 1. Based on GB/T16773-2008, the size of the coal fines in the proximate analysis was less than 1 mm in diameter. From Table 1, we can see that the *R*_max_ values of ZGZ and XZY are 1.17 and 0.85, respectively, which means the coal from the two coal mines is bituminous coal. The moisture values of the ZGZ and XZY coals were 8.3% and 2.3% respectively, and the carbon contents of the ZGZ and XZY coals were 53.82% and 68.47% respectively, meaning that the XZY coal is higher metamorphic. (Answer to question 3 reviewer 1).

**Table 1:** Results of proximate analysis and maceral of different samples.

Sample Name	Stromal vitrinite	Homogeneous vitrinite	Structural vitrinite	Mass vitrinite	*R*_min_/%	*R*_max_/%	Clay Mineral (%)	Moisture (%)	Ash yield (%)	Carbon (%)
ZGZ	7.60	49.30	15.70	4.70	1.10	1.17	1.00	8.30	14.70	53.82
XZY	19.50	11.00	41.20	3.70	0.76	0.85	1.50	2.30	1.61	68.47

## Results and Analysis

### NA and CA experimental results

The parameters obtained from the NA and CA experiments are shown in Table 2. The surface area of the coal samples was obtained by the Brunauer-Emmet-Teller method and the Dubinin–Radushkevich method. The average value of the specific surface area from the Zhao Ge Zhuang mine is 1.798 times that of the coal samples from the Xin Zhou Yao mine. In Table 2, the average porosity of coal samples from Zhao Ge Zhuang and Xin Zhou Yao mine are 5.69% and 10.79%. The maximum relative error between the average porosity and porosities of coal samples from Xin Zhou Yao mine is 16%. There is an apparent difference among the porosities of Zhao Ge Zhuang coal samples. For example, the porosity of ZGZ-6 coal sample is 3 times of ZGZ-5 coal sample, because the content of macropores in ZGZ-6 coal sample is much more than that in ZGZ-5 coal sample. This is not an indicator of poor repeatability of the measurements. Since coal’s heterogeneous physical property, coal samples from the same mine are possibly different numerically. (Answer to question 2 reviewer 1 and editor).

**Table 2:** Parameters obtained from the above experiments.

Sample ID	Skeletal density (g/cm^3^)	Porosity (%)	Pore diameters from N_2_ injection				Pore diameters from CO_2_ adsorption			Parameters obtained from MIP experiments		
			*S*_BET_ m^2^/g	*V*_BJH_ cm^3^/g	*V*_trans_ cm^3^/g	*V*_micro_ cm^3^/g	*S*_DR_ m^2^/g	*V*_s−m_ cm^3^/g	*V*_a−s_ mmol/g	Median pore diameters (nm)	Average pore diameters (nm)	BET surface area (m_2_/g)
ZGZ-1	1.032	7.258	3.400	0.009	0.003	0.001	121.096	0.002	0.052	9.400	8.400	2.269
ZGZ-2	1.201	7.368	1.124	0.002	0.000	0.001	68.112	0.005	0.115	18.600	18.600	3.400
ZGZ-3	1.080	2.850	1.723	0.002	0.001	0.001	74.716	0.005	0.100	22.500	12.200	1.124
ZGZ-4	1.200	6.326	2.269	0.005	0.003	0.001	99.800	0.003	0.790	10.500	12.100	0.216
ZGZ-5	1.011	2.568	2.006	0.007	0.004	0.001	45.178	0.004	0.090	9.200	8.200	0.101
ZGZ-6	1.160	7.770	2.387	0.007	0.004	0.001	64.711	0.004	0.098	12.700	9.700	2.006
XZY-1	1.237	10.185	0.885	0.001	0.001	0.001	54.169	0.001	0.018	56.600	15.000	4.002
XZY-2	1.390	11.126	0.617	0.002	0.001	0.001	78.660	0.013	0.073	51.900	14.400	4.960
XZY-3	1.296	12.274	0.726	0.002	0.001	0.001	89.702	0.016	0.100	56.100	14.400	4.297
XZY-4	1.387	10.600	0.846	0.003	0.002	0.001	120.639	0.021	0.559	51.600	12.300	0.846
XZY-5	1.401	11.250	2.645	0.007	0.002	0.002	117.545	0.019	0.550	34.700	11.100	2.645
XZY-6	1.395	9.296	1.462	0.005	0.003	0.001	105.151	0.018	0.515	29.400	10.600	1.462

Nomenclature: *S*_BET_, specific surface area acquired by Brunauer-Emmet-Teller method; *V*_BJH_, total pore volume calculated by Barrett Joyner Halenda method; *V*_trans_, transition pore volume (volume of pores with a diameter of 10–100 nm) of *V*_BJH_; *V*_micro_, micropore volume (volume of pores with a diameter of 2–10 nm) of *V*_BJH_; *S*_DR_, specific surface area obtained by Dubinin–Radushkevich method; *V*_s−m_, super-micropore volume (volume of pores with a diameter less than 2 nm); *V*_a−s_, volume of adsorbed CO_2_ amount in super-micropores.

**Figure 1:**
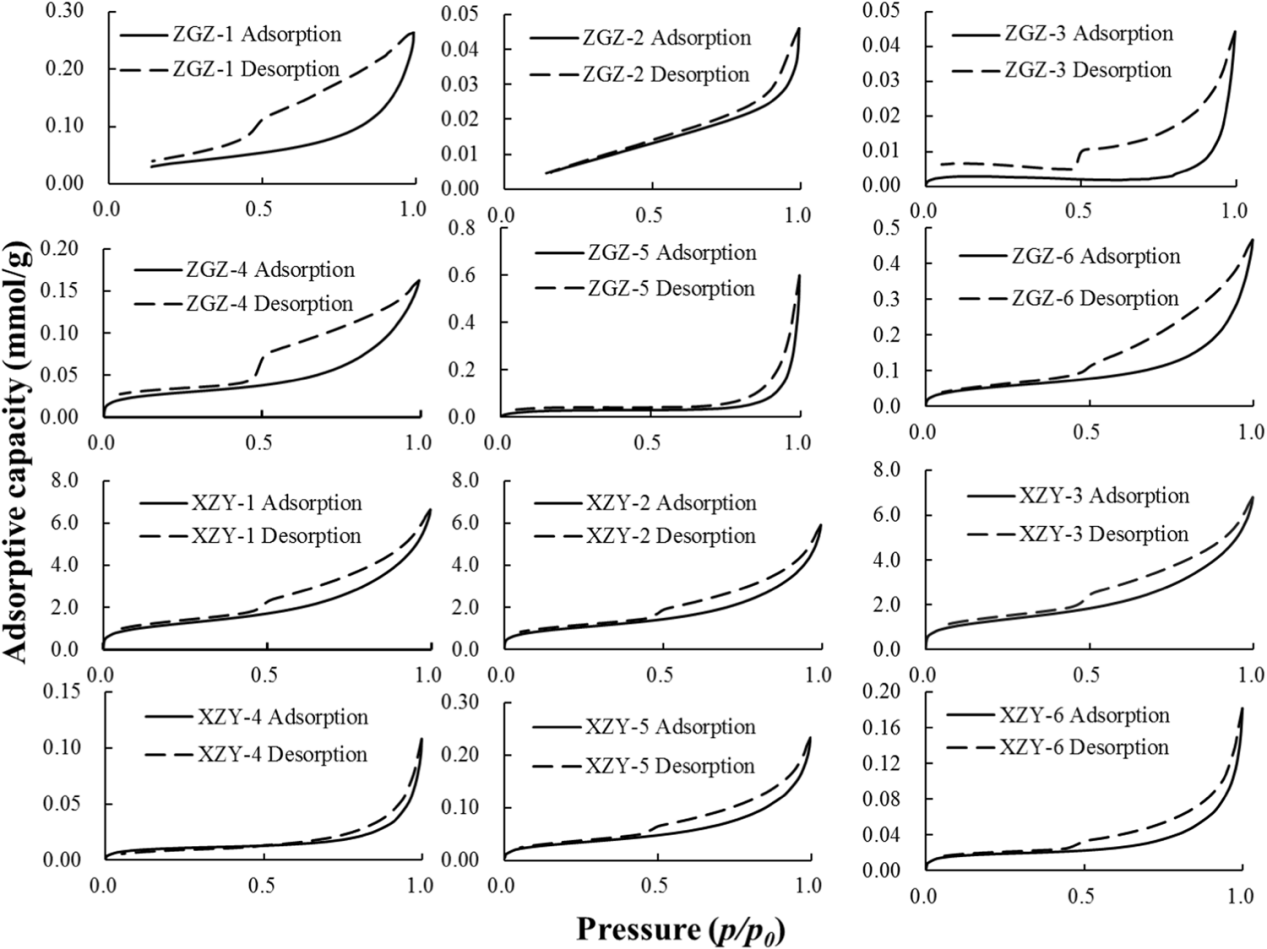
The isothermal adsorption/desorption curves of NA (*p*/*p*_*0*_ ≤ 1).

The isothermal adsorption/desorption curves of NA testing were obtained as shown in Fig. 1. As the relative pressure (*p*/*p*_*0*_) increases, the adsorption amount increases. In Fig. 1, the area of the desorption curve that deviates from the adsorption curve is called a hysteresis loop. According to previous studies of hysteresis loops^28,29^, the pore characteristics of coal samples can be obtained.

In the NA experiment, as the relative pressure increases, the amount of coal sample gas adsorption also increases. In the relative pressure interval of 0.2–0.7, the adsorption curve increases in a straight line, indicating that the coal sample has certain pores in the corresponding diameter range. The adsorption loop is narrow where the relative pressure is below 0.5 and wide where it is above 0.5, which indicates that the coal sample has open pores in a larger diameter range and some pores that are closed at one end in a smaller diameter range.

The adsorption isotherm shows an apparent increasing trend within a relative pressure greater than 0.8, which implies that the majority of the pores in the samples are smaller pores. The hysteretic loops of ZGZ-1 and ZGZ-6 are wider in the range of relative pressures greater than 0.5 and narrower for relative pressures less than 0.5, which indicates that the airtight pore is closed at one end in a range of smaller pore diameters and open pores in an interval of larger ones. When the relative pressure of the isotherms of ZGZ-1, ZGZ-3 and ZGZ-5 are approximately 0.5, there is a steep decrease on the desorption curve, which actually corresponds to ink-bottle pores. When the relative pressure is approximately 0.5, the desorption curves of the five Xin Zhou Yao coal samples (except for XZY-4) show a steep decrease, which belongs to the adsorption loop corresponding to the ink-bottle pores. The features of the desorption curves of the XZY coal samples show that ink-bottle pores exist in a smaller pore diameter range and open pores in larger one.

### MIP experimental results

**Figure 2:**
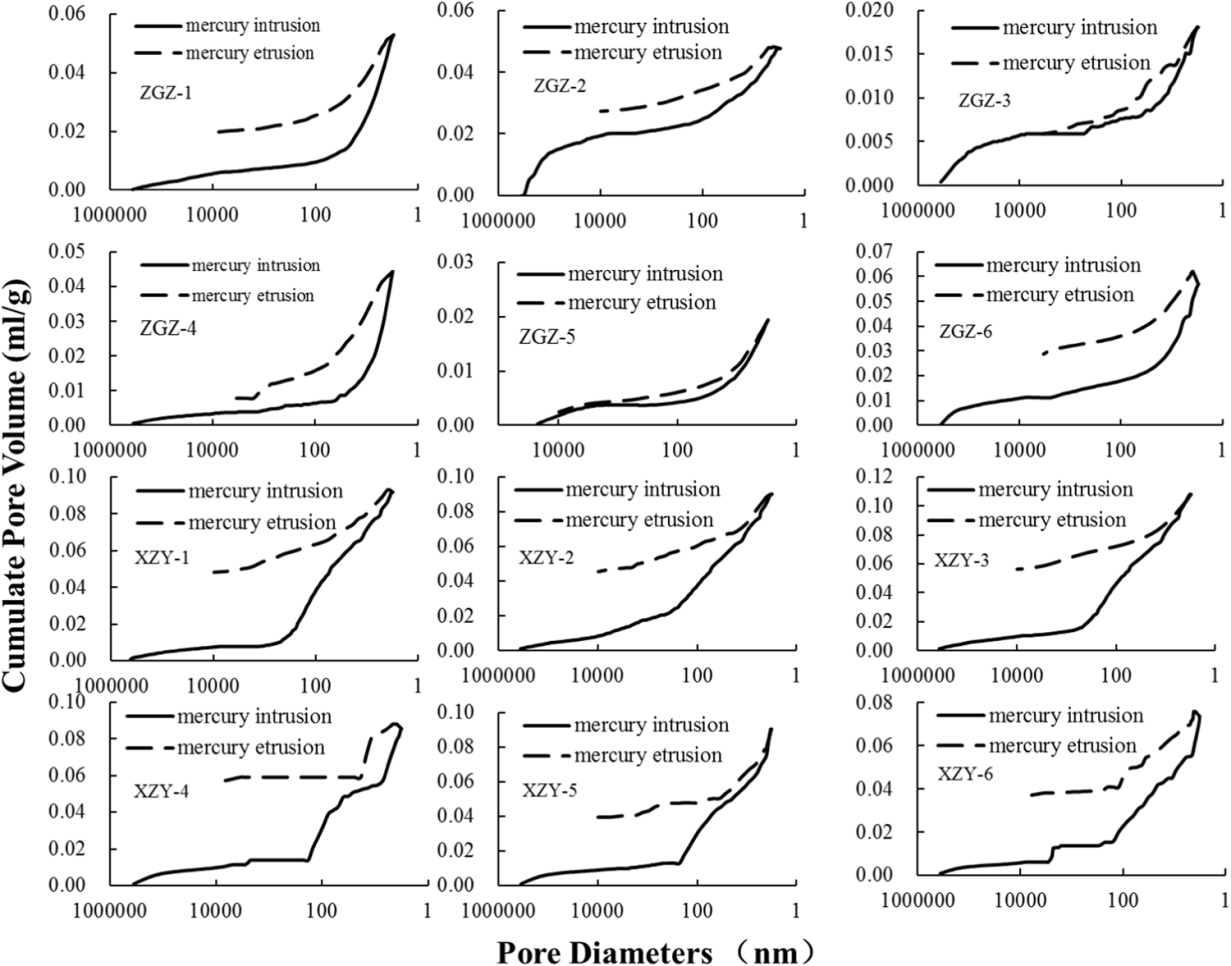
Mercury intrusion porosimetry curve.

The parameters obtained from the MIP experiments are listed in Table 2. It is shown that the average porosity, median pore diameter, average pore diameter and surface of the coal samples from the Xin Zhou Yao mine are more than those of the samples from the Zhao Ge Zhuang mine. The MIP curves are shown in Fig. 2. Most MIP curves for the ZGZ samples (except for ZGZ-1 and ZGZ-4) are “S” shaped because of an increase of mercury intrusion at the beginning. This result illustrates that these samples have many pores with diameters of more than 10000 nm. However, the MIP curves of the XZY samples are roughly “L” shaped, which means that with increases in the Hg pressure, there is a certain amount of corresponding pores. The proportion of macropores in the XZY samples is less than that of the ZGZ samples.

In this paper, the pore characteristics of the coal samples are obtained by analyzing the mercury curves of the coal samples and the hysteresis loops between the mercury injection and the ejection curves. During the mercury injection experiment, the area between the mercury injection and the ejection curves is called the mercury injection hysteresis loop and is formed by the existence of open pores in the coal samples. These open pores result in mercury not being ejected during the process of decompression. Figure 2 shows the mercury injection/ejection curves and the mercury hysteresis loops of the coal samples. The coal samples from the Xin Zhou Yao mine have a relatively wide mercury hysteresis loop, indicating that there are more open pores in these coal samples than in the ZGZ coal samples. The mercury hysteresis loops of the ZGZ coal samples are relatively narrow, which indicates that the samples mainly contain semi-open pores. The mercury curves are gentle overall, but the mercury ejection curves of samples ZGZ-4, XZY-4 and XZY-6 show steep decreases, which means that the ZGZ-4, XZY-4 and XZY-6 coal samples have ink-bottle pores inside. A jump in mercury intrusion and extrusion curves of the XZY 4, 5, 6 coal samples exist, and this is a normal phenomenon during the pressure transition from low to high. (Answer to question 5 reviewer 1).

### Pore size distribution

Because the coal matrix is a compressible material, the influence of coal compressibility on the volume of instruction mercury cannot be overlooked. It has been shown in previous studies that the compressibility of the coal matrix could have an effect on the mercury quantity during the MIP process^24,30^. The highest mercury intrusion pressure is approximately 400 MPa under which the coal matrix is significantly compressed. The compressibility of the coal matrix, *K*_c_ is defined as^24^

1 $${K}_{c}=\frac{d{V}_{c}}{{V}_{c}dp}$$

*V*_*c*_ is the coal matrix volume, and $\frac{d{V}_{c}}{dp}$ is the function of the coal matrix changing with pressure. The compressibility of the coal matrix has been calculated to be between 8.6067 × 10^−5^ MPa^−1^ and 13.1691 × 10^−5^ MPa^−1^, which agrees with the results of Toda and Toyoda^31^.

As a result of the limitations of experimental conditions, high pressures may compress the coal matrix and damage narrow pores. Therefore, it is necessary to combine the data of MIP and NA to calculate the compressibility of the coal matrix simultaneously.

The volume change of the mercury instruction experiment can be defined as *ΔV*_*obs*_:

2 $$\varDelta {V}_{obs}=\varDelta {V}_{p}+\varDelta {V}_{c}$$

**Figure 3:**
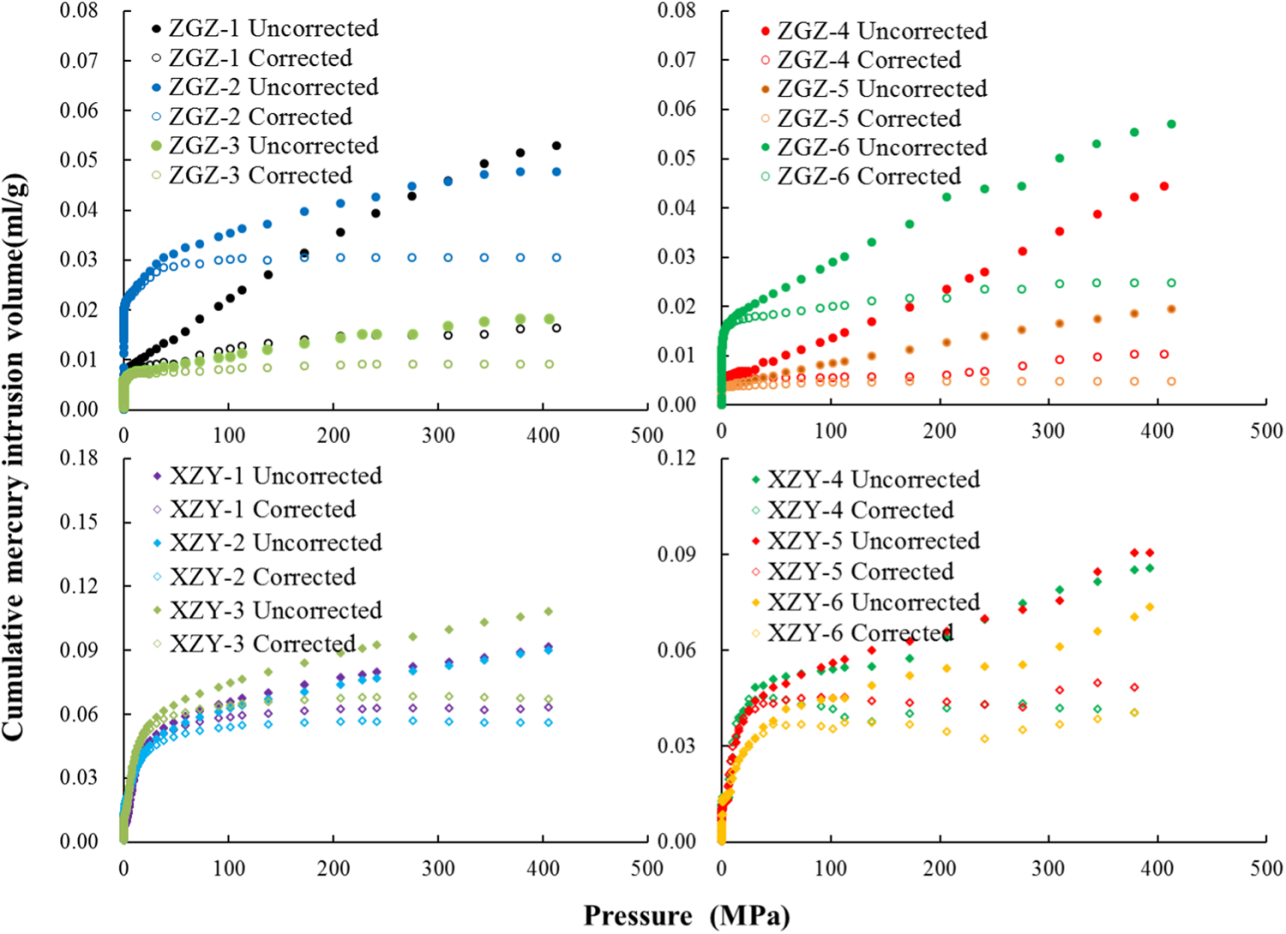
The cumulative mercury intrusion volume before and after correction.

*ΔV*_*p*_ is the volume of pore filling, and *ΔV*_*c*_ is the compression volume of the coal matrix. As seen in Fig. 3, when the pressure is higher than 10 MPa, there is roughly a linear relationship between the volume of mercury instruction and pressure. Hence, it can be supposed that when the pressure is over 10 MPa, *ΔV*_*obs*_*/ΔP* is a constant defined as *β*. *ΔV*_*c*_/*ΔP* can be defined as^24^

3 $$\frac{\Delta {V}_{c}}{\Delta {V}_{p}}=\beta -\frac{{\sum }_{{R}_{min}}^{{R}_{max}}\,\Delta {V}_{p}}{\Delta P}$$

It can be assumed that $\frac{\Delta {V}_{c}}{\Delta p}$ is independent of pressure, and $\frac{\Delta {V}_{c}}{\Delta p}$ can replace $\frac{dVc}{dp}$:

4 $${K}_{c}=(\beta -\frac{{\sum }_{{R}_{min}}^{{R}_{max}}\,\Delta {V}_{p}}{\varDelta P})/{V}_{c}$$

The value of compressibility obtained by Eq. (4) agrees with previous studies^24,31–33^. Among these, *R*_max_ and *R*_min_ are the maximum and minimum pore diameters, respectively, corresponding to the pore volume that needs to be corrected. Thus, the true coal matrix volume, *V*_*c*_, can be obtained by the true coal density and the sample mass. The mesopore volume and the super micropores are obtained from the NA data and the CA data, respectively. The cumulative mercury intrusion volume is adjusted in Fig. 3.

As seen in Fig. 3 and Table 3, the compressibility of the coal matrix has a distinct impact on the volume of mercury instruction. The corresponding pore size is a pore diameter less than 100 nm. This conclusion is consistent with the results of Song *et al*.^34^. The average corrected pore volume for the XZY samples is 0.053 ml/g, which is almost 3 times more than the average pore volume of the ZGZ samples.

**Table 3:** Compressibility correction parameters.

Sample ID	*k*_c_ × 10^−5^ MPa^−1^	*β* (10^−5^)	*R*^2^	Cumulative pore volume (ml/g)	Corrected umulative pore volume (ml/g)
ZGZ-1	9.9917	11.7	0.989556	0.0529	0.01632
ZGZ-2	10.8016	5.0	0.970900	0.0477	0.03039
ZGZ-3	11.2896	3.0	0.982700	0.0181	0.00913
ZGZ-4	13.1691	9.5	0.990700	0.0443	0.0102
ZGZ-5	10.0637	4.0	0.996522	0.0194	0.01559
ZGZ-6	12.0962	10.1	0.995500	0.0569	0.02479
XZY-1	8.6067	8.2	0.988390	0.0917	0.06319
XZY-2	11.6250	9.0	0.994000	0.0901	0.05578
XZY-3	13.2410	10.0	0.992000	0.1080	0.06692
XZY-4	11.7013	11.7	0.983144	0.0855	0.04041
XZY-5	11.7166	11.7	0.976296	0.0905	0.04836
XZY-6	8.9170	8.9	0.959792	0.0735	0.04044

**Figure 4:**
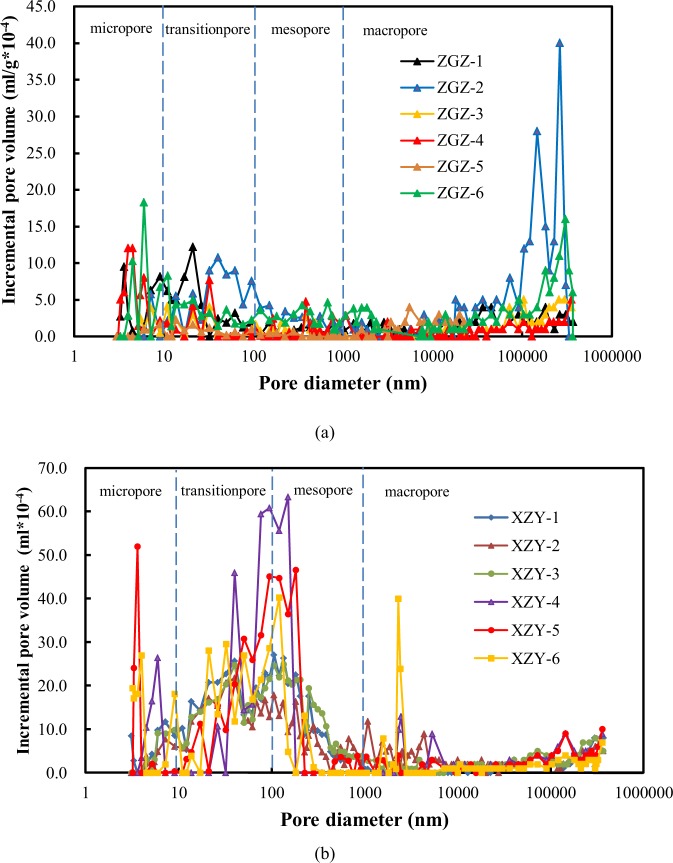
Pore size distribution of different coal samples (**a**) Pore size distribution of ZGZ-1~6 (**b**) Pore size distribution of XZY-1~6.

The PSD statistics of the coal samples after correction were obtained as shown in Fig. 4. The dominant pores of the XZY samples are almost transition pores and mesopores, unlike the main pores of the ZGZ samples, which are more macropores and micropores. In general, pores with diameters greater than 100 nm are known as seepage pores^35^. In this work, the mean percentage of the seepage pores in the XZY samples is 37.7%, which is higher than that of the ZGZ samples (30.2%). Based on the pore features, the pore structure of the XZY coal samples makes gas seep and flow more easily, while the pores of the ZGZ samples do not.

## Discussion

### Multifractal characteristics of coal samples

As previous studies^36^ showes, multifractals are an extension and a superposition of monofractals, which means, geometrically, multifractal structures can be decomposed into a set of intertwined fractal subsets that characterize the local variability and heterogeneity of the studied variables. (Answer to Question 5 reviewer 2). Based on the corrected MIP data, multifractal parameters can be calculated. There are two equivalent mathematical ways to describe the multifractal characterization: multifractal singularity spectra (*α* ~ *f*(*α*))^37,38^ and generalized fractal dimension spectra (*q* ~ *D*(*q*))^31^.

To use the multifractal singularity spectra, there are three parameters should be calculated: the probability quality distribution *P*_*i*_ (*ε*), the singular exponent *α* and the dimension function *f* (*α*). To determine the parameter probability quality distribution *P*_*i*_ (*ε*), the box dimension method is used to analyze the mercury data. According to the dichotomy principle^39^, the entire range corresponding to all the MIP data is divided into *N* subintervals so that the length of each interval is equal. Above N = 2^k^, the k value is theoretically preferable to any real number greater than or equal to 0. To ensure that each box is not empty, the value of k is 6. In the interval division, the aperture variation range (3 nm-400 μm) is too large, which is not convenient to choose; therefore, the interval is transformed logarithmically. The length of the transformed interval is *I*^26^

5 $$I=log({d}_{max}/{d}_{min})$$

Then, combining the MIP data, it is easy to obtain the corresponding mercury injection of each interval, *N*_*i*_. Therefore, we can calculate the mass probability *P*_*i*_.

6 $${P}_{i}(\varepsilon )=\frac{{N}_{i}(\varepsilon )}{{N}_{t}}$$

*N*_*i*_ is the content of mercury in each small box, and *N*_*t*_ is the final content of mercury obtained after the modified mercury injection.

The relationship between the quality probability *P*_*i*_ and the interval length *ε* is as follows:

7 $${P}_{i}(\varepsilon )={\varepsilon }^{{\alpha }_{i}}$$

*α* is the local singular parameter of the mass probability function *P*_*i*_, describing the inhomogeneity of the probability subset.

To facilitate research by amplifying the areas of high probability (high values) and those of low probability (low values), this study uses the concept of the statistical moment order, *q*, which can be applied from(−∞, +∞). In this paper, we take the *q* value into −10, 10 with each step length of 0.5. When *q* > 1, the high value information is amplified, and the low value information is reduced, whereas the opposite holds when *q* < −1. Thus, the new probability measure, *ω*, is constructed successfully^37^.

8 $$\varOmega (q,\varepsilon )=\frac{{P}_{i}^{q}(\varepsilon )}{{\sum }_{i=1}^{N(\varepsilon )}\,{P}_{i}^{q}(\varepsilon )}$$

After calculation, the pore size distributions of the coal samples show good linear fitting between the logarithm of *ω*(*q*, *ε*) and *ε*. The values of the fitting coefficient *R*^2^ are all greater than 0.9, which is consistent with the results of Vidal Vázquez E^40^. Thus, it can be concluded that the pore size distributions of the coal samples have multifractal characteristics.

The curve composed of *α* and *f*(*α*) is called the multifractal singular spectrum. In the single fractal, *α* is a constant and a point in the multifractal spectrum. For multiple fractals, the multifractal spectrum is a single peak convex function of *α*^41^. The two parameter values are obtained as follows^38,42^:

9 $$\alpha (q)\propto \frac{{\sum }_{i=1}^{N(\varepsilon )}\,{\omega }_{i}(q,\varepsilon )\,{\rm{lg}}\,[{P}_{i}(\varepsilon )]}{{\rm{lg}}(\varepsilon )}$$

10 $$f[\alpha (q)]\propto \frac{{\sum }_{i=1}^{N(\varepsilon )}\,{\omega }_{i}(q,\varepsilon )lg[{\omega }_{i}(q,\varepsilon )]\,}{lg(\varepsilon )}$$

**Figure 5:**
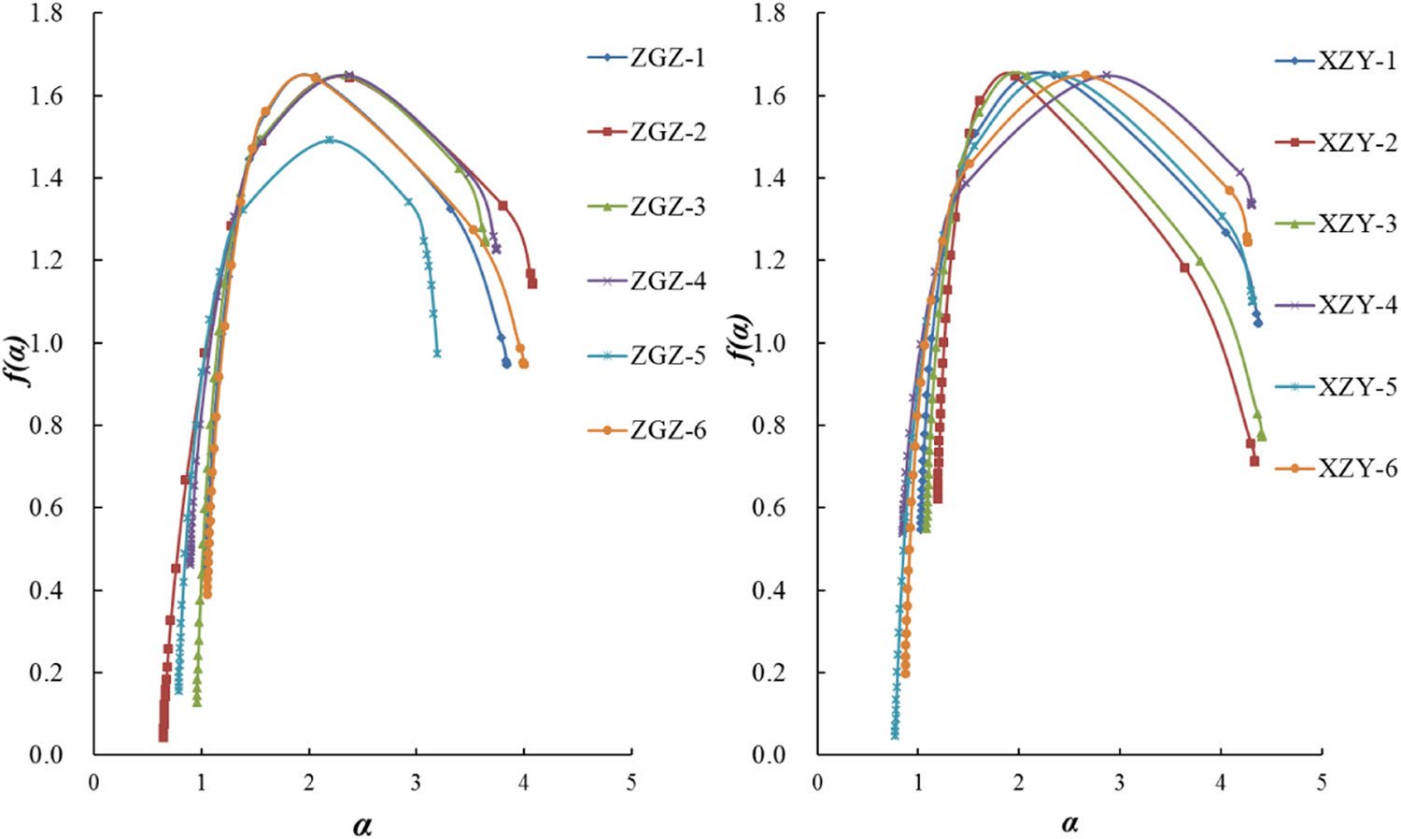
Multifractal singular spectrum of the samples.

The corresponding *α* and *f*(*α*) values can be calculated from Eqs. (9 and 10), and the multifractal singular spectrum is shown in Fig. 5. According to the multifractal theory^25^, the characteristic parameters of the *f*(*α*) spectrum and the singular spectrum can be analyzed, which can reflect the inhomogeneity of the fractal distribution of the internal pore volume of the coal samples. By studying the width of the *f*(*α*) spectrum, the pore distribution complexity of the coal samples can be analyzed. With the increase of the width of the *f*(*α*) spectrum, the complexity of the pore size distribution increases. As shown in Fig. 5, the width of the *f*(*α*) spectra of the XZY samples is wider than that of the ZGZ samples, indicating that the XZY coal samples have a higher spatial heterogeneity and complexity in their pore size distribution.

The concentration of the pore volume distribution can be obtained by studying the singular fractal index *α*. The higher the value of *α*_*0*_ for a coal sample is, the higher the heterogeneity of the distribution of the pore volume in the corresponding coal sample is, and the more apparent the fluctuation is^24^. As shown in Table 4, the average of the multiple singular fractal index, *α*_*0*_, of the Xin Zhou Yao and ZGZ coal samples is 2.397 and 2.236, respectively. This result indicates that the distribution inhomogeneity of the inner pore diameter of the coal samples from the 9# coal seam (coal and gas outburst seam) in the Zhao Ge Zhuang mine is lower than that of the 11# coal seam (bump-prone coal seam) in the Xin Zhou Yao mine, the pore size distribution of the XZY samples is denser in certain intervals, and the distribution area in the matrix is narrow.

**Table 4:** The calculated parameters of the multifractal singular spectrum and the generalized fractal dimension spectrum.

Sample ID	*α*_0_	*α*_*q*+_	*α*_*q*−_	*α*_0_ − *α*_*q*+_	*α*_0_ − *α*_*q*−_	*α*_*q*−_ − *α*_*q*+_	*H*	*D*_0_	*D*_1_	*D*_2_	*D*_10_	*D*_−10_	*D*_0_ − *D*_10_	*D*_0_ − *D*_−10_	*D*_−10_ − *D*_10_
ZGZ-1	2.066	1.042	3.842	1.025	1.042	2.800	1.172	1.649	1.446	1.344	1.112	3.579	0.536	1.93	2.466
ZGZ-2	2.384	0.651	4.084	1.733	0.651	3.433	1.023	1.643	1.282	1.046	0.718	3.816	0.924	2.173	3.098
ZGZ-3	2.340	0.955	3.642	1.385	0.955	2.687	1.139	1.649	1.366	1.279	1.047	3.424	0.601	1.775	2.376
ZGZ-4	2.377	0.898	3.746	1.478	0.898	2.848	1.079	1.649	1.306	1.157	0.947	3.517	0.702	1.868	2.570
ZGZ-5	2.191	0.788	3.376	1.403	0.788	2.588	1.041	1.649	1.172	1.081	0.858	3.071	0.633	1.58	2.212
ZGZ-6	2.061	1.057	4.000	1.004	1.057	2.943	1.185	1.643	1.47	1.369	1.131	3.722	0.511	2.08	2.591
XZY-1	2.355	1.030	4.376	1.326	1.030	3.347	1.126	1.65	1.358	1.252	1.083	4.073	0.567	2.484	2.991
XZY-2	1.960	1.196	4.388	0.765	1.196	3.143	1.216	1.647	1.508	1.431	1.261	4.008	0.386	2.361	2.748
XZY-3	2.083	1.088	4.409	0.996	1.088	3.321	1.166	1.647	1.437	1.332	1.148	4.078	0.499	2.431	2.931
XZY-4	2.867	0.854	4.306	2.014	0.854	3.453	1.02	1.649	1.172	1.039	0.889	4.036	0.760	2.387	3.147
XZY-5	2.457	0.776	4.315	1.681	0.776	3.539	1.051	1.649	1.259	1.102	0.857	4.022	0.791	2.374	3.165
XZY-6	2.660	0.873	4.271	1.787	0.873	3.397	1.069	1.649	1.246	1.138	0.948	3.995	0.700	2.347	3.047

In this work, the *α*_*0*_ of the coal samples can be listed in descending order: XZY-4 > XZY-6 > XZY-5 > ZGZ-2 > ZGZ-4 > XZY-1 > ZGZ-3 > ZGZ-5 > XZY-3 > ZGZ-1 > ZGZ-6 > XZY-2. Among these values, the singular fractal index of XZY-4 is much higher than that of ZGZ-2, with a difference of 0.484. The average value of *α*_*0*_ of the coal samples from the Xin Zhou Yao mine is 1.072 times that of the coal samples from the Zhao Ge Zhuang mine. This difference shows that the fluctuation of the pore distribution of the XZY coal samples is greater than that of the ZGZ coal samples, which is consistent with the previous analysis results of the fluctuation variations of *α*_*0*_.

In the multifractal singular spectrum, the left and right sides divided by the vertical line *α* = *α*_*0*_ represent different variable information. *α*_*q*_ − *α*_*0*_ (right peak) is defined as the low value information (the sparse part of the pore distribution), and the opposite *α*_*0*_* − α*_*q*+_ (left peak) is defined as the high value information (the denser part of the pore distribution). If the width of the left side of the peak is greater than that of the right part, the high value information has a significant influence on the pore size distribution. Inversely, the low-value information has a significant influence on the pore size distribution. Except for ZGZ-1, ZGZ-6, XZY-2 and XZY-3, the width of the right side of the coal samples is more than that of the left side, so the pores with smaller pore diameters dominate the PSDs.

Table 4 shows that the values of *α*_*q*_ − *α*_*q*+_ for the coal samples from the Xin Zhou mine are 1.20~1.07 times the maximal values of the samples from the Zhao Ge Zhuang mine, which indicates a more complex pore space distribution for the Xin Zhou Yao coal samples.

### The generalized fractal dimension spectrum of coal samples

The generalized fractal dimension (*q* − *D*_*q*_) was used to obtain more accurate and comprehensive pore characteristics of the coal samples. Similarly, in the generalized fractal measurement, using *P*_*i*_ (*ε*)^*q*^ to highlight the local influence^37^,

11 $$\mathop{\sum }\limits_{i=1}^{N(\varepsilon )}\,{P}_{i}{(\varepsilon )}^{q}={\varepsilon }^{(q-1){D}_{q}}$$

where *q* takes a step length of 0.5, and the value range is −10, 10. The length of each box equals to *ε* taken in the box dimension method, and *N* (*ε*) is the number of boxes.

The generalized fractal dimension *D*_*q*_ was defined by Grassberger and Procaccia^36^ and derived from Eq. (11), which is expressed as

12 $${D}_{q}=\frac{1}{q-1}{li}{{m}}_{\varepsilon \to 0}\frac{log{\sum }_{i=1}^{N(\varepsilon )}\,{P}_{i}{(\varepsilon )}^{q}}{log(\varepsilon )}$$

when *q* ≠ 1, *the D*_*q*_ value calculation is suitable for Eq. (12), but when *q* = 1, *D*_*q*_ cannot be directly calculated. According to L’Hopital’s law, *D*_*1*_ can be obtained and is equal to *f*(*α*_(*1*)_). As a result, the value of *D*_*1*_ can be calculated by using Eq. (13)^37,38^.

13 $$F[\alpha (q)]={\mathrm{lim}}_{\varepsilon \to 0}\frac{{\sum }_{i=1}^{N(\varepsilon )}\,{\omega }_{i}(q,\varepsilon )lg[{\omega }_{i}(q,\varepsilon )]\,}{lg(\varepsilon )}\,$$

The dimension *D*_*0*_ is called the capacity dimension, *D*_*1*_ is the information dimension and *D*_*2*_ is referred to as the correlation dimension^41^. When, the calculated *D*_*q*_ mainly reflects the high porosity region in the coal samples, as *q* < 0, the value of *D*_*q*_ especially expresses the area of low porosity in the coal samples.

Meanwhile, the Hurst exponent can be calculated as^43^

14 $$H=({D}_{2}+1)/2$$

The Hurst exponent is usually associated with positive autocorrelation or long-range spatial variation^44^. The value of H (Hurst exponent) has also been proven to be an effective parameter to estimate the pore connectivity of different coals^22^. The average H value of the XZY and ZGZ coal samples is 1.106 and 1.108, respectively, which indicates a slightly higher pore connectivity in the XZY coal samples.

**Figure 6:**
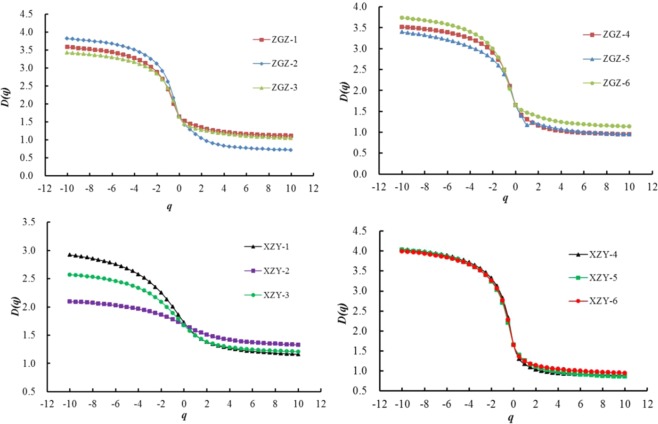
Generalized fractal dimension spectrum.

The generalized fractal dimension spectrum is obtained, as shown in Fig. 6. Meanwhile, the generalized fractal parameters are listed in Table 4. In the generalized fractal dimension spectrum, the heterogeneity of the porosity can be reflected by the width of *D*_−*10*_ − *D*_*10*_, *D*_*0*_ − *D*_*10*_ and *D*_−*10*_ − *D*_*0*_. There is a positive correlation between the heterogeneity for the pore volume distribution along the pore size distribution and the width of *D*_−*10*_ − *D*_*10*_. The right side of the general fractal dimension spectrum *D*_*0*_ − *D*_*10*_ and the left side *D*_−*10*_ − *D*_*0*_ correspond to the dominance of high and low concentrations of porosity, respectively^26,44^.

The minimum value of *D*_−*10*_ − *D*_*10*_ is found for the ZGZ-5 coal sample, and the maximum value of 3.165 is found in the XZY-5 sample. This result shows that ZGZ-5 has the smallest heterogeneity of pore volume distribution of different pore diameters, while XZY-5 has the largest heterogeneity. According to the multi-fractal analysis of the 12 coal samples, the PSD heterogeneity of the XZY coal samples is significantly higher than that of the ZGZ coal samples. The average *D*_−*10*_ − *D*_*10*_ value of the coal samples from the Xin Zhou Yao mine is 1.18 times the average value of the ZGZ samples. Therefore, the PSDs of the coal samples from the Xin Zhou Yao mine are more heterogeneous than those of the coal samples from the Zhao Ge Zhuang mine. The results are consistent with the analysis of the singular fractal dimension spectrum.

*D*_*0*_ and *D*_*1*_ are called the capacity and information dimensions, respectively, and the closer *D*_1_ is to *D*_0_, the more evenly distributed the porosity is. For the samples from the Xin Zhou Yao and Zhao Ge Zhuang mines, the averages of the difference between *D*_1_ and *D*_0_ are 0.319 and 0.307, respectively. Thus, the pore size distribution of XZY coal samples is more heterogeneous. Caniego indicated that the smaller *D*_1_ is, the higher the heterogeneity is^37^. The mean value of *D*_1_ for the ZGZ coal samples is 1.34, which is higher than that from the Xin Zhou Yao mine. This result also indicates a higher heterogeneity for the XZY coal samples.

## Conclusions

In this paper, the PSDs of coal samples from coal bump and gas outburst coal seams are investigated by mercury intrusion porosimetry, nitrogen adsorption and carbon dioxide adsorption. Based on the multifractal theory, the pore fractal characteristics are also discussed. The main conclusions can be drawn as follows:

1. The compressibility of the coal matrix has an important influence on the pore volume measured by the MIP experiments, especially when the mercury pressure is greater than 20 MPa. To systematically assess the pore structure, it is necessary to correct the MIP results by combining the MIP, NA and CA data. The experimental results show that open pores, semi-open pores, and ink-bottle pores exist in both groups of coal samples.
2. The dominant pores of the coal samples from the coal and gas outburst coal seam are mainly micropores with pore diameters of less than 10 nm and macropores with diameters greater than 100 μm. The main pores of the coal samples from the bump-prone coal seam are transition pores (diameters of 10–100 nm) and mesopores (diameters of 100–1000 nm). The porosity, especially the seepage pore content, of the bump-prone coal samples is higher than that of the coal and gas burst coal samples.
3. The multifractal singular spectrum and the generalized fractal dimension spectrum of the coal samples indicate that the coal samples from the different coal seams have a strong autocorrelation in the size-dependent distribution of porosity and diverse PSD characteristics. Compared with the samples from the Zhao Ge Zhuang mine (gas outburst coal seam), the coal samples from the Xin Zhou Yao mine (bump-prone coal seam) have a higher heterogeneity and pore connectivity.
